# Rapidly progressive Kaposi’s sarcoma associated with human immunodeficiency syndrome^☆^

**DOI:** 10.1016/j.abd.2021.12.008

**Published:** 2022-11-26

**Authors:** Camila Gonçalves Pinheiro, Francisco de Assis Silva Paiva, Isabelle Sousa Medeiros Torres Ferreira, Gabriela Teixeira Viana Suppa Meira, Antônio Carlos Evangelista de Araújo Bonfim, Luciana Cavalcante Trindade

**Affiliations:** a Dermatology Service, Nova Esperança Faculty of Medicine, João Pessoa, PB, Brazil; b Infectology Service, Complexo Hospitalar Clementino Fraga, João Pessoa, PB, Brazil; c Centro de Diagnóstico Anatomopatológico, João Pessoa, PB, Brazil; d Dermatology Service, Complexo Hospitalar Clementino Fraga, João Pessoa, PB, Brazil

Dear Editor,

In Acquired Immunodeficiency Syndrome (AIDS) there is a deficiency of T-cell-mediated immunity, making the host vulnerable to opportunistic infections and malignancies.^1^ It occurs when the number of CD4 cells is <200 mm^3^ or in the presence of some defining condition, such as Kaposi sarcoma (KS), which is the most frequent neoplasm in these patients with diagnosis based on clinical and histopathological findings.^2^, ^3^

The present report describes a 31-year-old male patient who presented with a violaceous lesion on the oral cavity, which progressed in size and extension within two months, associated with intense odynophagia. He reported having sex with men. On physical examination, he had a violaceous, infiltrated tumor with areas of leukoplakia, which occupied about 70% of the hard palate (Fig. 1). In addition, he had non-painful purpuric lesions, violaceous papules, nodules and tumors on the face, scalp, cervical region, axillae and trunk, oval in shape and of different sizes (Figure 2, Figure 3). Serological tests confirmed HIV and syphilis infection. The CD4 lymphocyte count was 129 mm^3^ at the time of the diagnosis. Histopathology of a violaceous skin lesion showed an atypical vascular proliferation affecting the dermis and on immunohistochemistry positivity for CD31 and herpes virus 8 (HHV-8), confirming the diagnosis of KS (Fig. 4). Chest radiography and upper digestive endoscopy were performed, which showed no changes suggestive of malignancy. Antiretroviral therapy (ART) and systemic chemotherapy were started during hospitalization, with initial improvement of the lesions and overall condition. In the follow up, a new CD4 cell count was requested three months after the start of ART, disclosing a value of 320 cells/mm^3^, demonstrating improvement in relation to the first test. However, after approximately eight months, the patient returned to the Infectious Diseases Unit with respiratory distress and hematemesis. He was transferred to the ICU of the Oncology Unit, with rapid progression to death without due clarification of the cause of the bleeding.

**Figure 1:**
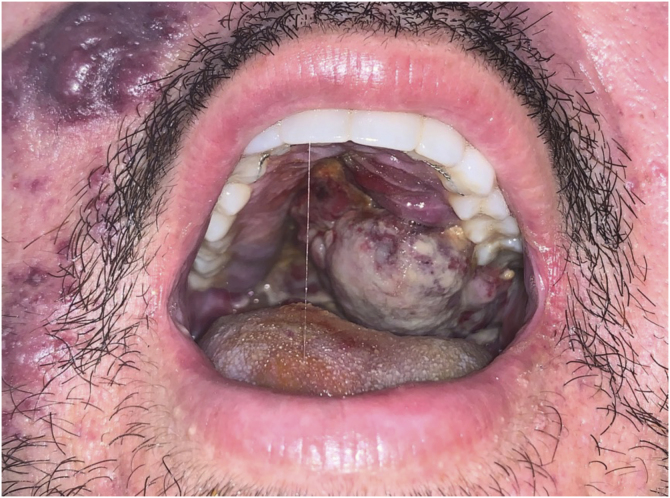
Infiltrated violaceous tumor and areas of leukoplakia on the palate.

**Figure 2:**
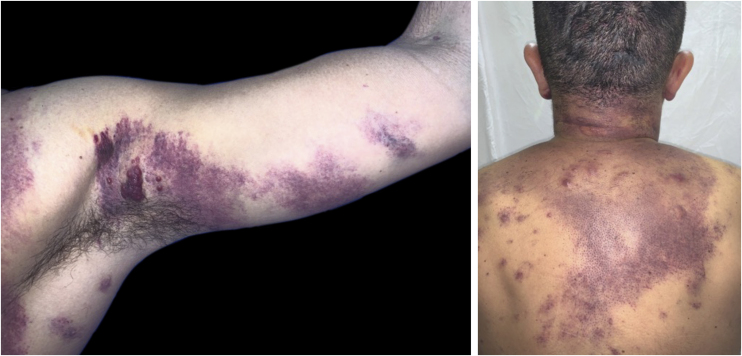
Non-painful purpuric lesions, violaceous papules, nodules and tumors of varying sizes on the armpit, upper limb, cervical region and trunk.

**Figure 3:**
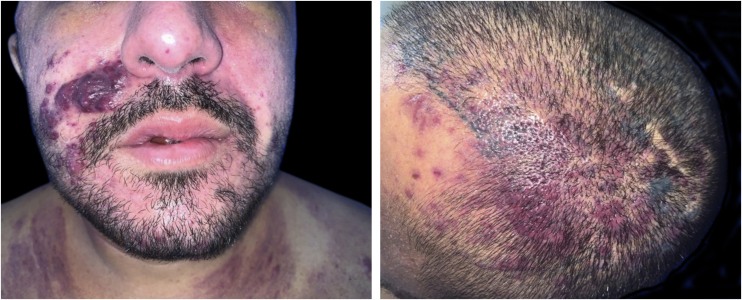
Non-painful papules, nodules and violaceous tumors on the face and scalp, of varying sizes.

**Figure 4:**
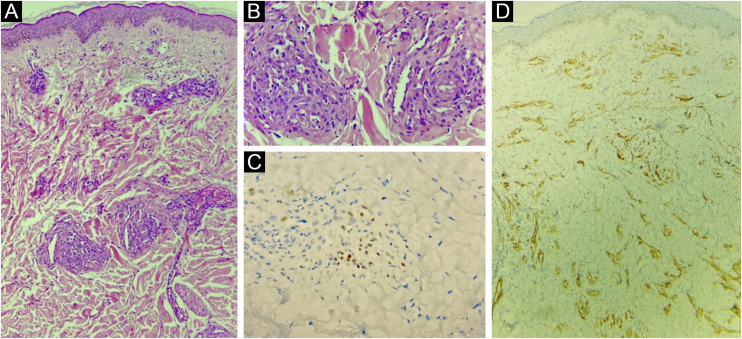
(A) Atypical vascular proliferation, with irregular vessels and endothelial atypia (Hematoxylin & eosin, low power). (B) Detail of the vascular neoplasia (Hematoxylin & eosin, ×100). (C) Immunohistochemistry of the skin fragment showing positivity for human herpes virus 8 (HHV8). (D) Immunohistochemistry of the skin fragment showing positivity for the endothelial cell marker CD31.

KS is an endothelial malignancy and the epidemic clinical form is associated with HIV infection. Its causes and risk factors include herpesvirus type 8 infection, immunosuppression, genetic predisposition, and the presence of HLA DR5.^4^, ^5^

It may be the first manifestation of AIDS and indicates late diagnosis. The clinical manifestations of KS include asymptomatic violaceous, erythematous or brownish macules that progress to papules, plaques or tumors, which may bleed or ulcerate. There may be concomitant involvement of the gastrointestinal tract, lungs, lymph nodes, bones, and liver.^4^

On histopathology, the initial lesions show proliferation and dilation of the dermal vessels, with large endothelial cells; perivascular infiltrate of lymphocytes and plasma cells, extravasated erythrocytes and hemosiderin deposits. In the plaque and nodular stage, there is a proliferation of blood vessels and atypical spindle cells.^6^ There is also immunoreactivity with endothelial cell markers, such as CD34 and CD31.

After the implementation of ART in AIDS, there has been a reduction in the frequency of KS.^5^ Drugs have led to the regression of cutaneous and visceral lesions, possibly through direct anti-angiogenic effects and immune restoration.^7^ The decrease in KS cases may also be due to the lower number of AIDS cases among homosexual/bisexual men since this neoplasm characteristically affects this population.^8^

Treatment includes both local and systemic therapies and depends on factors such as the KS subtype, the course of the disease, its extent, and the patient's symptoms. The management of localized lesions includes radiotherapy, surgical excision, cryosurgery and laser. If there are multiple skin lesions and/or visceral involvement, chemotherapy is indicated. As for the HIV-related KS subtype, ART is the first treatment option.^4^ The prognosis and evolution of KS are related to immunosuppression and the presence of opportunistic infections.

Despite the reduction in AIDS detection rate in Brazil in recent years,^9^ sexual intercourse remains the main transmission route among men. Thus, health education and ongoing debates on the topic remain relevant to encourage prevention and an early diagnosis of HIV infection.

## Financial support

None declared.

## Authors' contributions

Camila Gonçalves Pinheiro: Collection of data, drafting of the manuscript.

Francisco de Assis Silva Paiva: Collection of data.

Isabelle Sousa Medeiros Torres Ferreira: Collection of data.

Gabriela Teixeira Viana Suppa Meira: Collection of data.

Antônio Carlos Evangelista de Araújo Bonfim: Collection of data.

Luciana Cavalcante Trindade: Critical review to obtain intellectual content, and approval of the final version of the manuscript.

## Conflicts of interest

None declared.
